# Association between polymorphisms of DNA repair genes and intracranial aneurysms: A systematic review and meta‑analysis

**DOI:** 10.3892/mi.2024.183

**Published:** 2024-07-24

**Authors:** Mohamed M. Montasr, George Fotakopoulos, Vasiliki Epameinondas Georgakopoulou, Ourania Fotakopoulou, Nikolaos Trakas, Pagona Sklapani, Kostas N. Fountas

**Affiliations:** 1 Department of Neurosurgery, General University Hospital of Larissa, 41221 Larissa, Greece; 2 Department of Pathophysiology, National and Kapodistrian University of Athens, 11527 Athens, Greece; 3 Department of Pediatrics, General Hospital of Zakynthos ‘Agios Dionysios’, 29100 Zakynthos, Greece; 4 Department of Biochemistry, Sismanogleio Hospital, 15126 Athens, Greece

**Keywords:** intracranial aneurysms, aneurysms, gene associations, single nucleotide polymorphisms, chondroitin sulfate proteoglycan 2 gene

## Abstract

Intracranial aneurysms (IAs) are present in ~2% of the general population, and genetic factors cannot be excluded for the risk of their development. The gene factors that result in the changes in the vascular extracellular matrix (ECM) may also be a key reason for IAs being hereditary. The *VCAN* gene [also known as chondroitin sulfate proteoglycan 2 (*CSPG2*)] plays various roles in maintaining ECM functions. The present systematic review and meta-analysis aimed to investigate all eligible articles involving IAs on the association with germ line SNPs of DNA repair genes (up to January, 2024). The total number of patients was 2,308 [987 cases (poor outcomes) and 1,321 controls (good outcomes)]. The results revealed that rs2287926 G/G genotype and G allele and rs251124 T/T genotype and minor allele T increased the risk of developing IAs. However, further studies are required to examine these gene polymorphisms as screening markers for IAs.

## Introduction

Intracranial aneurysms (IAs) can be found in ~2% of the general population and are revealed in 85% of subarachnoid hemorrhage cases (1-3). More commonly, patients between 40 and 60 years of age are more prone to IA rupture with an unfavorable outcome. It has been found that ~50% of cases succumb and another 20% remain with disability after management (4-7).

Several investigators have inspected the expansion of IAs, which are usually considered to be caused by the interactivity among environmental and hereditary causes (8-10). For example, environmental factors, such as alcohol consumption, smoking and hypertension are suggested to be common risk factors for IAs (8-10). On the other hand, hereditary causes cannot be eliminated as a risk factor for IAs. Hereditary susceptibility is the main risk parameter for the formation of IA, with an up to seven-fold increased risk of rupture (11). Another study also stated a five-fold increased risk of IA rupture in a first-degree individual among families with a prevalence of IAs (12). Furthermore, genes cause alterations in the vascular extracellular matrix (ECM), which may also be a significant cause for IAs to be hereditary entities (13,14). ECM modification (disruption or remodeling) plays a crucial role in preserving the composition and integrity of the arterial wall. ECM degradation is a key characteristic of IAs (13,14).

The Versican (*VCAN*) gene [also known as chondroitin sulfate proteoglycan 2 (*CSPG2*)] has multiple functions in preserving ECM roles (13,14). Previous research has indicated that the three single nucleotide polymorphisms (SNPs), rs2287926, rs173686 and rs251124 of the *VCAN* gene, are related to the development of IAs (3,13,14). Nevertheless, replication research has stated a lack of an association among the *CSPG2* variants (rs251124 and rs173686) and the presence of aneurysms (15,16).

The purpose of the present meta-analysis was to evaluate the involvement of the *VCAN* gene variants in the development of IAs and its importance as a screening global marker for the pathogenesis of IAs. The findings presented herein may help clinicians identify the exact risk of their development.

## Data and methods

### Study identification and selection

**Figure 1:**
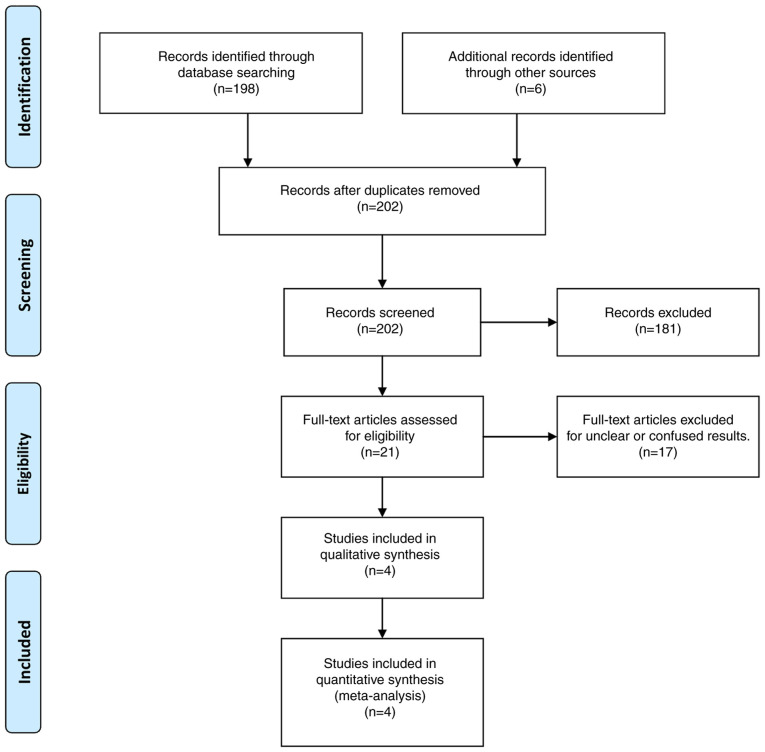
Flowchart of the study selection process.

To investigate all eligible articles involving IAs in the literature written in the English language on the association with germ-line SNPs of DNA repair genes and IAs through electronic databases, including the Cochrane Library, PubMed (up to January, 2024), Embase (up to January, 2024) and MEDLINE (up to January, 2024) via combinations of the following terms: Intracranial aneurysms; aneurysms; gene associations; SNPs; *CSPG2* gene. For the study protocol creation and design, the Preferred Reporting Items for Systematic Reviews and Meta-Analyses (PRISMA) guidelines were applied. The flowchart with the collection process of the enclosed articles is illustrated in Fig. 1. The present study integrated articles published between 1980 and 2023.

The subsequent information was inserted from the article pool: The authors' names and year of publication, the sample of total cases and controls, the mean age and sex of cases and controls, the DNA polymorphisms and DNA repair genes inspected in the study, genotyping techniques, the polymorphism's genotype, the number of cases and controls, the chromosome position, and the possible mechanisms of function were considered qualified. For the *CSPG2* rs188703, rs2287926, rs251124 and rs173686 SNPs, meta-analyses were carried out (Table I). Of note, data from genome-wide association studies were not included in any phases, but only data from candidate gene association studies were included. All genetic models (dominant, recessive and additive) were evaluated in studies recording genotype frequencies. The alleles and the SNP IDs are presented in Table I.

### Statistical analysis and assessment of heterogeneity

All analyses were performed using Review Manager Software (RevMan), version 5.4 (https://training.cochrane.org/onlinelearning/coresoftware/revman). A fixed- and random-effects model meta-analysis was used [according to the Cochrane Handbook for Systematic Reviews of Interventions (version 5.1.0) (17)] for the evaluation of the proportion estimate for every outcome independently, as the I^2^ statistic calculated the heterogeneity. A value of I2 in an amount <50% was considered as low heterogeneity, and a value >50% as high heterogeneity. Or else, the fixed-effect model was performed. The continuous outcomes were stated as a weighted mean difference with 95% confidence intervals (CIs). For discontinuous variables, odds ratios (ORs) with 95% CIs were used for the evaluation. A P-value <0.05 was considered to indicate a statistically significant difference.

## Results

### Included articles

In total, four articles (15,16,18,19) fulfilled the eligibility criteria. The total number of patients was 2,308 [987 cases (poor outcomes) and 1,321 controls (good outcomes)]. The study sample was based on four studies (15,16,18,19) (Table II).

### Epidemiological and clinical features

The authors, publication year, genes, SNP ID, mean age of the patients, and sample of males and females between the cases and controls among the studies are presented in Table II.

### CSPG2-rs188703

**Figure 2:**
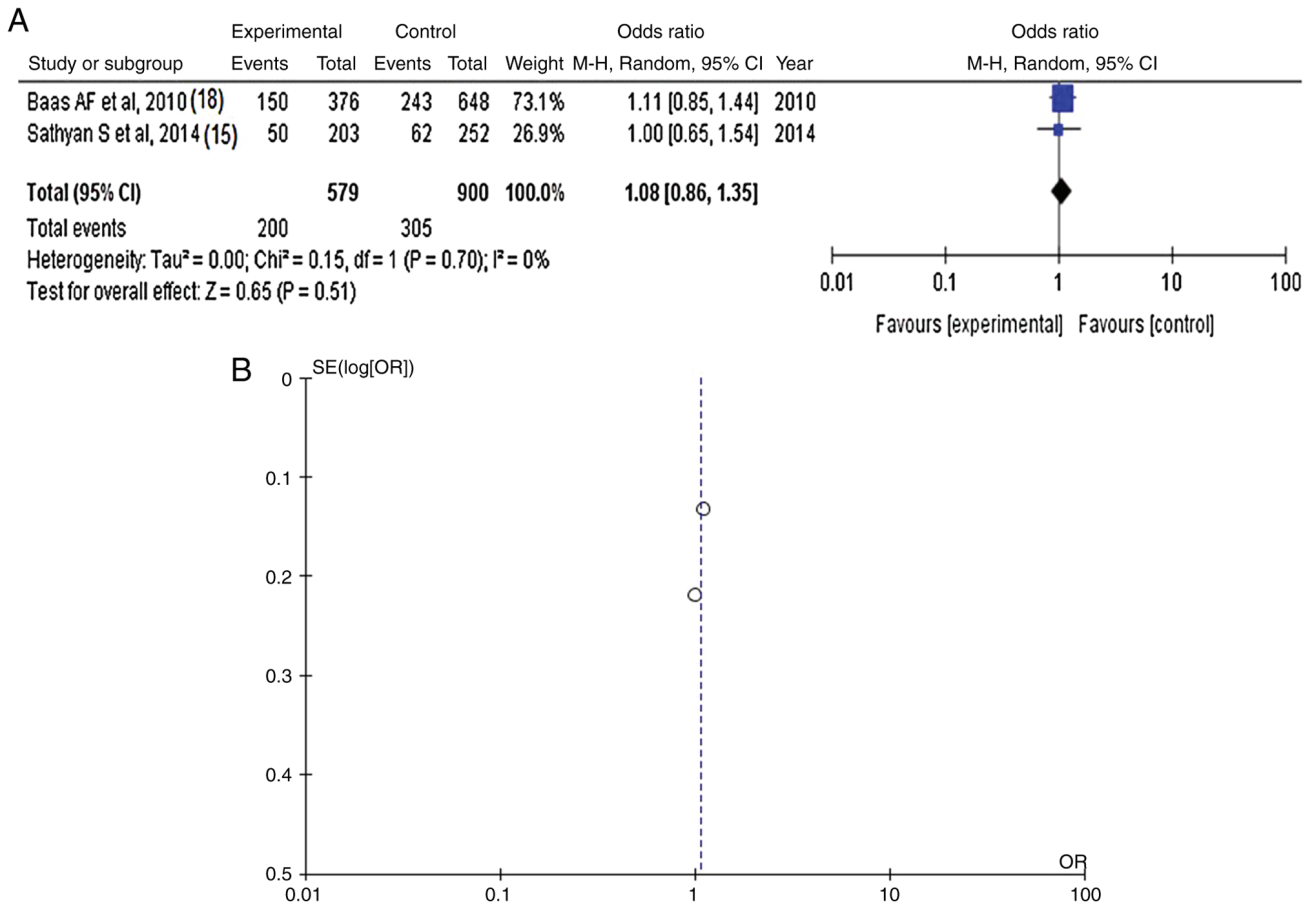
(A) Forest plot for *CSPG2*-rs188703. The results demonstrate no statistically significant difference between patients with intracranial aneurysms (cases with poor outcomes) and the controls (OR, 1.08; 95% CI, 0.86-1.35; P=0.51), and without heterogeneity (P=0.70 and I^2^=0%). (B) Funnel plots of the *CSPG2*-rs188703 with no heterogeneity (P=0.70 and I^2^=0%) and thus, no publication bias. *CSPG2*, chondroitin sulfate proteoglycan 2; I^2^, the percentage of total variation across studies that is due to heterogeneity rather than chance; CI, confidence interval; P, P-value; OR, odds ratio.

Information regarding the SNP rs188703 was available in two articles (15,18) and demonstrated no statistically significant difference between the patients with IAs (cases with poor outcomes) and the controls (OR, 1.08; 95% CI, 0.86-1.35; P=0.51) (Fig. 2A and Table III). SNP rs188703 was found in 305 of 900 (33.8%) control patients and in 200 of 579 (34.5%) cases. When investigating the funnel plot, it was established that there was no heterogeneity (P=0.70 and I^2^=0%) and no publication bias (Fig. 2B).

### CSPG2-rs2287926

**Figure 3:**
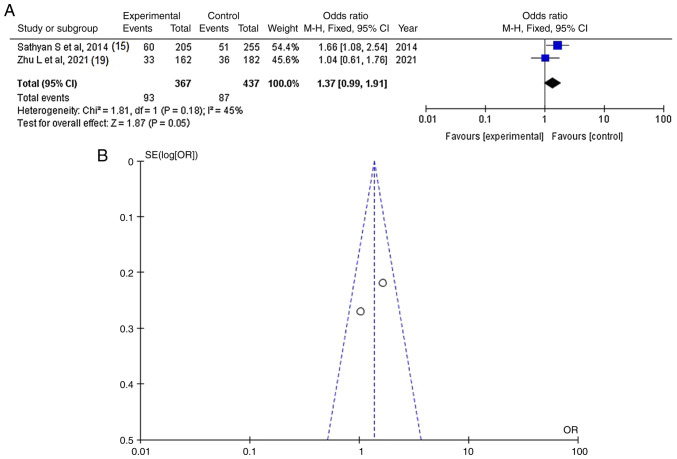
(A) Forest plot for *CSPG2*-rs2287926. The results demonstrate a statistically significant difference between patients with intracranial aneurysms (cases with poor outcomes) and the controls (OR, 1.37; 95% CI, 0.99-1.91; P=0.0500), and with low heterogeneity (P=0.18 and I^2^=45%). (B) Funnel plots of the *CSPG2*-rs2287926 with low heterogeneity (P=0.18 and I^2^=45%) and thus, low publication bias. *CSPG2*, chondroitin sulfate proteoglycan 2; I^2^, the percentage of total variation across studies that is due to heterogeneity rather than chance; CI, confidence interval; P, P-value; OR, odds ratio.

As regards SNP rs2287926, information was available in two articles (15,19) and demonstrated a statistically significant difference between the patients with IAs (cases with poor outcomes) and the controls (OR, 1.37; 95% CI, 0.99-1.91; P=0.0500) (Fig. 3A and Table III). SNP rs2287926 was found in 87 of 437 (19.9%) control patients and in 93 of 367 (25.3%) cases. When investigating the funnel plot, it was established that there was low heterogeneity (P=0.18 and I^2^=45%) and low publication bias (Fig. 3B).

### CSPG2-rs251124

**Figure 4:**
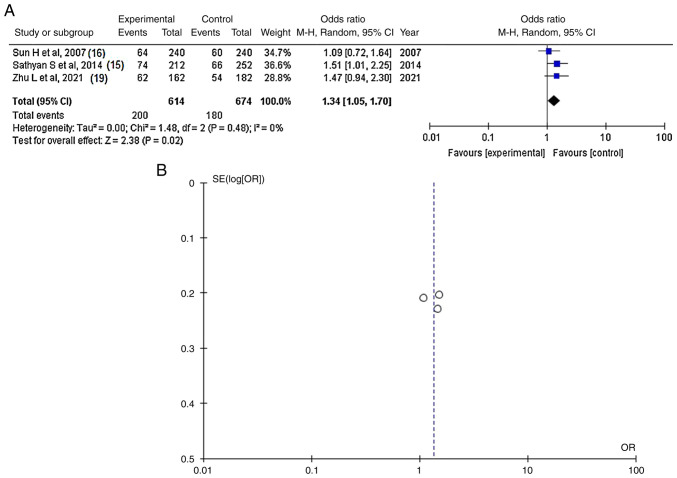
(A) Forest plot for *CSPG2*-rs251124. The results demonstrate a statistically significant difference between patients with intracranial aneurysms (cases with poor outcomes) and the controls (OR, 1.34; 95% CI, 1.05-1.70; and P=0.02), and with no heterogeneity (P=0.48 and I^2^=0%). (B) Funnel plots of the *CSPG2*-rs251124 with no heterogeneity (P=0.48 and I^2^=0%) and thus, no publication bias. *CSPG2*, chondroitin sulfate proteoglycan 2; I^2^, the percentage of total variation across studies that is due to heterogeneity rather than chance; CI, confidence interval; P, P-value; OR, odds ratio.

Information regarding the SNP rs251124 was available in three articles (15,16,19) and demonstrated a statistically significant difference between the patients with IAs (cases with poor outcomes) and the controls (OR, 1.34; 95% CI, 1.05-1.70; P=0.02) (Fig. 4A and Table III). SNP rs251124 was found in 180 of 674 (26.7%) control patients and in 200 of 614 (32.5%) cases. When investigating the funnel plot, it was established that there was no heterogeneity (P=0.48 and I^2^=0%) and no publication bias (Fig. 4B).

### CSPG2-rs173686

**Figure 5:**
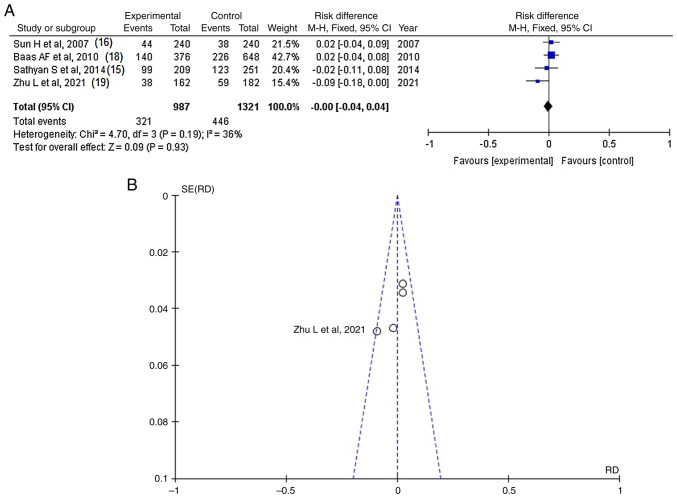
(A) Forest plot for *CSPG2*-rs173686. The results demonstrate no statistically significant difference between patients with intracranial aneurysms (cases with poor outcomes) and the controls [OR, -0.00; 95% CI, (-0.04)-0.04; P=0.93], and with low heterogeneity (P=0.19 and I^2^=36%). (B) Funnel plots of the *CSPG2*-rs173686 with low heterogeneity (P=0.19 and I^2^=36%) and thus, low publication bias. *CSPG2*, chondroitin sulfate proteoglycan 2; I^2^, the percentage of total variation across studies that is due to heterogeneity rather than chance; CI, confidence interval; P, P-value; OR, odds ratio.

As regards SNP rs173686, information was available in four articles (15,16,18,19) and demonstrated no statistically significant difference between the patients with IAs (cases with poor outcomes) and the controls [OR, -0.00; 95% CI, (-0.04)-0.04; and P=0.93] (Fig. 5A and Table III). SNP rs173686 was found in 446 of 1,321 (33.7%) control patients and in 321 of 987 (32.5%) cases. When investigating the funnel plot, it was established that there was low heterogeneity (P=0.19 and I^2^=36%) and low publication bias (Fig. 5B).

## Discussion

There are different hypotheses about the formation of aneurysms, most of which involve inappropriate ECM modification (19). The *VCAN* gene plays several roles in preserving ECM processes (13,14). It has been shown that individuals with the rs251124 polymorphism T allele are at an increased risk of developing IAs, and the CT genotype has been found to be distinct as a risk factor for the incidence of aneurysms in a southern Indian population (20). Overall, there has been an association between the development of IAs in the European population and the rs173686 polymorphism (19). Specifically, it has been shown that the rs173686 G/G genotype and G allele are associated with an increased risk of IA formation (13,19). The present meta-analysis investigated the associations among four SNPs in the *VCAN* gene polymorphism and IA susceptibility. The results revealed that the rs2287926 G/G genotype and G allele, and rs251124 T/T genotype and minor T allele increased the risk of developing IAs. No significant differences in allele or genotype frequencies among the control and case groups were noted with the rs188703 and rs173686 SNPs. Additional results revealed the *VCAN* gene to be involved in the development of IAs.

In the literature, it has been mentioned that the CT genotype is involved in the evolution of aneurysms in a few populations (19). Additionally, there is no evidence of an association between the rs173686 polymorphism in both genotypic and allelic levels and IA in the South Indian population (15). The present meta-analysis, based on a pool of 2,308 cases, identified the minor T allele of the rs251124 T/T genotype and the rs2287926 G/G genotype and G allele.

In the literature, researchers have verified the association between VCAN (CSPG2) genes and the risk of developing IAs (13,14). On the contrary, according to certain studies, there is no confirmed association between the CSPG2 variants (rs173686 and rs251124 polymorphisms) and IA susceptibility (3,16,19). These studies report that geographical and racial reasons may affect the aforementioned conflicting results. However, the heterogeneity of the population and the small number of cases may be the reasons for these different results compared with the present meta-analysis.

In addition, similar to the results of previous studies, members of the same ethnic group also have diverse susceptibilities (3,14,15). Sun *et al* (16) reported that the two rs25112 and four rs173686 SNPs of the CSPG2 gene were not associated with IAs in the Northern Chinese (Beijing) Han population. It is known that the incidence of IAs may be the consequence of complex results from both hereditary and environmental aspects. The primary explanation for this discrepancy may be topographical variations (19). Other environmental reasons, including sex, cigarettes and alcohol consumption, hypertension and hormonal profiles have been proposed to be associated with the development of IAs (9,10,21,22). On the other hand, ECM-related genes can also undergo alterations from various environmental factors, such as radiation, provoking a remodeling or disruption of the ECM of the arterial wall (13).

The present study had certain limitations which should be mentioned. The present meta-analysis pool was performed from proportionately small samples; consequently, the results require authentication from a large-scale participant. Another limitation was the heterogeneity between populations. It has been hypothesized that hereditary heterogeneity between different populations can lead to paradoxical results. In addition, the majority of enrolled articles included patients with ruptured and unruptured IAs; thus, aneurysm rapture may not be associated with SNP variation.

In conclusion, the present meta-analysis revealed that rs2287926 G/G genotype and minor allele G, rs251124 T/T genotype and minor T allele were associated with an increased risk of developing IAs. The results presented herein suggest that the *VCAN* (CSPG2) gene is an IA-predisposed gene and merits further investigation as a screening global marker for IAs. Thus, *VCAN* is a key candidate gene involved in the pathogenesis of IAs. This may help clinicians indentify the exact risk factors associated with the development of IAs. Future studies are required however, to examine these gene polymorphisms as screening markers for IAs.

## Figures and Tables

**Table I:** Gene, chromosome position, possible mechanisms of function, and number of studies included in the present meta-analysis.

Gene	Allele (SNP ID)	SNP	Chromosome position	Possible mechanisms of function	Total no. of studies included
*CSPG2*	G>A (rs188703)	R1826H	5q14.2	protein_coding splicing_regulation transcriptional_regulation post_translation	2
*CSPG2*	G>A Additive (rs2287926)	G428D	5q14.2	protein_coding splicing_regulation transcriptional_regulation post_translation	2
*CSPG2*	C>T Additive (rs251124)	(Gly428Asp)	5q14.2	transcriptional_regulation	3
*CSPG2*	A>G Additive (rs173686)	-	5q14.2	transcriptional_regulation	4

*CSPG2*, chondroitin sulfate proteoglycan 2 (Versican); SNP, single nucleotide polymorphism.

**Table II:** Design and baseline characteristics of the trials included in the present meta-analysis.

							Cases, sex		Controls sex		
Authors, year of publication	Gene	SNPs ID	Cases Sample	Controls Sample	Cases Mean age	Controls, Mean age	M	F	M	F	(Refs.)
Sun *et al*, 2007	*CSPG2*	rs173688 and rs251126	240	240	45.14±11.66	41.82±8.96	104	136	116	124	(16)
Baas *et al*, 2010	*CSPG2*	rs173689 and rs188703	376	648	72.1	-	337	39	-	-	(18)
Sathyan *et al*, 2014	*CSPG2*	rs173687, rs251125, rs2287926 and rs188703	220	250	51.17±11.37	51.0±14.1	123	97	122	128	(15)
Zhu *et al*, 2021	*CSPG2*	rs173686, rs251124, and rs2287926	162	182	58.08±11.93	60.25±11.80	60	102	92	90	(19)

*CSPG2*, chondroitin sulfate proteoglycan 2 (Versican); SNP, single nucleotide polymorphism.

**Table III:** Results from the meta-analyses of the CSPG2 rs188703, rs2287926, rs251124 and rs173686 for association with IAs.

				Heterogeneity			Test for overall effect		
Gene	Polymorphism	No. of Included studies	Population	I^2^ (%)	P-value	Meta-analysis model	OR (95% CI)	P-value	(Refs.)
*CSPG2*	rs188703	2	Mixed	0	0.70	Random	1.08(0.86-1.35)	0.51	(15,18)
*CSPG2*	rs2287926	2	Mixed	45	0.18	Fixed	1.37(0.99-1.91)	**0.05**	(15,19)
*CSPG2*	rs251124	3	Mixed	0	0.48	Random	1.34(1.05-1.70)	**0.02**	(15,16,19)
*CSPG2*	rs173686	4	Mixed	36	0.19	Random	-0.00[(-0.04)-0.04)]	0.93	(15,16,18,19)

Values in bold font indicate a statistically significant difference (P<0.05). I^2^, the percentage of total variation across studies that is due to heterogeneity rather than chance; CI, confidence interval; *CSPG2*, chondroitin sulfate proteoglycan 2 (Versican); IAs, intracranial aneurysms.

## Data Availability

The datasets used and/or analyzed during the current study are available from the corresponding author on reasonable request.
